# Intravesical Foreign Body via a Vesicoperineal Fistula

**DOI:** 10.1155/2013/659582

**Published:** 2013-04-04

**Authors:** Aaron M. Potretzke, Kelvin S. Wong, Tracy M. Downs

**Affiliations:** Department of Urology, University of Wisconsin-Madison, 1685 Highland Avenue, Madison, WI 53705, USA

## Abstract

Male urethral “play” has been described for centuries. There are serious potential complications in this. We present a bizarre case of a variant of such play. A 49-year-old man presented with abdominal pain and incontinence. He had created a “neovagina” at the perineum for self-pleasure. The handle of a toilet brush was placed in the neovagina for self-pleasure but retracted into the bladder via a vesicoperineal fistula. An open cystotomy was performed to remove the foreign body.

## 1. Introduction

The artistic and written description of unusual genitourinary tract activities (UGUA) has been around for several centuries. Participation in such activities has been described across cultures and nations [1]. Contemporary case reports and reviews have focused on the embedment of fluids or foreign objects in the genitalia or placement into the urethra, that is, “urethral play.” Numerous reports have described peculiar objects being placed within the genitourinary tract, for example, battery, pencil, pearl, beads, light bulb, small milk carton, electrical wire, carrot, toy, magnet, dog penis, and a decapitated snake [1–5]. Most often the motivation for UGUA is sexual stimulation, but curiosity, psychological issues, and intoxication have also been linked [2].

The migration of foreign bodies into the bladder from surrounding anatomic structures has been reported, for example, intrauterine device, artificial urinary sphincter, prosthetic sling, vaginal pessary, and nonabsorbable suture [3]. To our knowledge, we present the first case of migration of a foreign body to the bladder from a self-inflicted perineal defect.

## 2. Case Presentation

**Figure 1:**
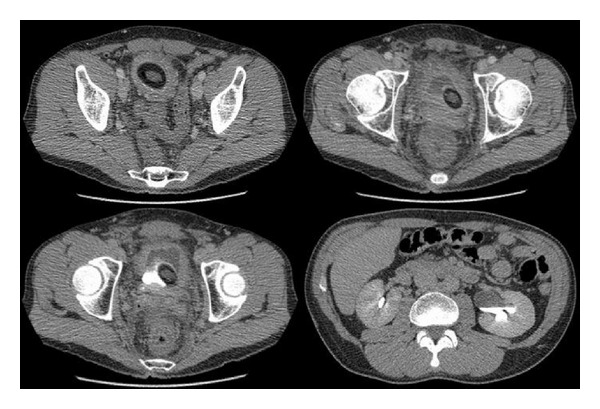
CT scan of the pelvis, demonstrating the toilet brush and surrounding fluid. There is a left-sided hydronephrosis (right, lower picture).

A 49-year-old man presented to the emergency room with complaints of lower abdominal pain and urinary incontinence. A CT scan was ordered (Figure 1). On urologic examination, a 6 cm self-induced incision was discovered longitudinally along the perineal raphe. Six years previously he had sharply incised his perineal raphe to create a “vagina.” He indicated that the impetus for self-mutilation was feelings of guilt that he had associated with his sexual abuse of a family member over twenty years before. However, he placed foreign objects in the created space in order to derive sexual pleasure. Two weeks previously, influenced by alcohol intoxication, he had inserted the handle of a toilet brush and was unable to retrieve it.

**Figure 2:**
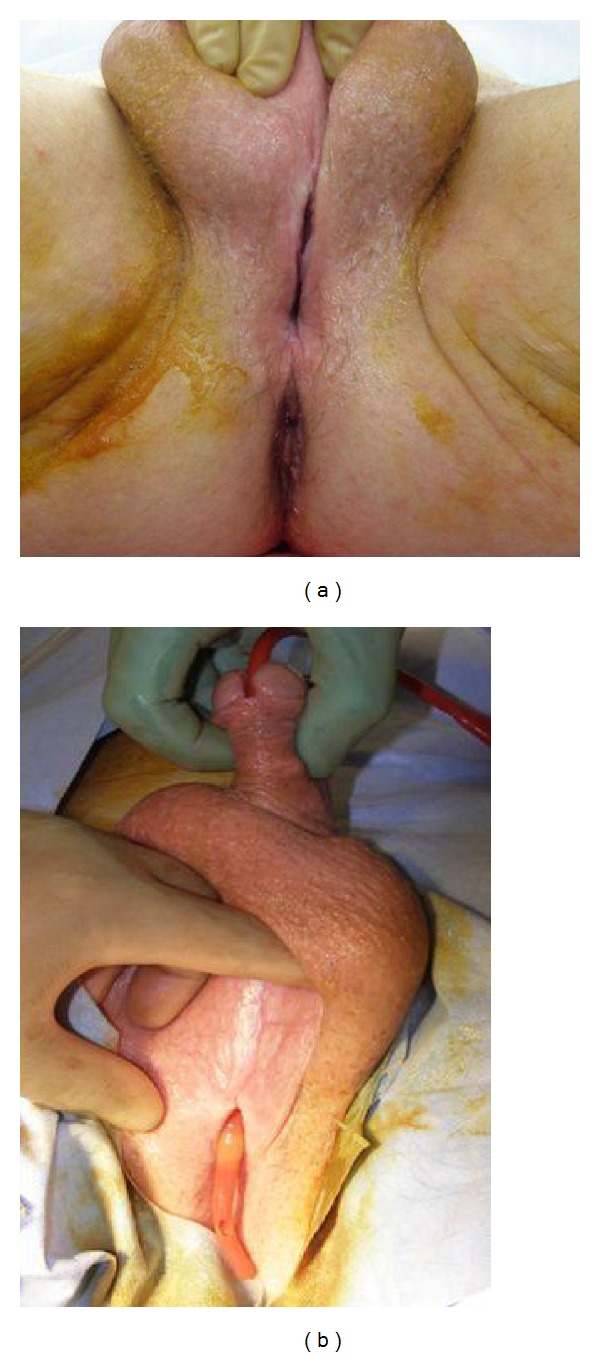
Intraoperative exam of the perineal defect (a). Coude-tipped foley catheter demonstrating the bulbar urethral-cutaneous fistula associated with the superior aspect of the perineal defect (b).

**Figure 3:**
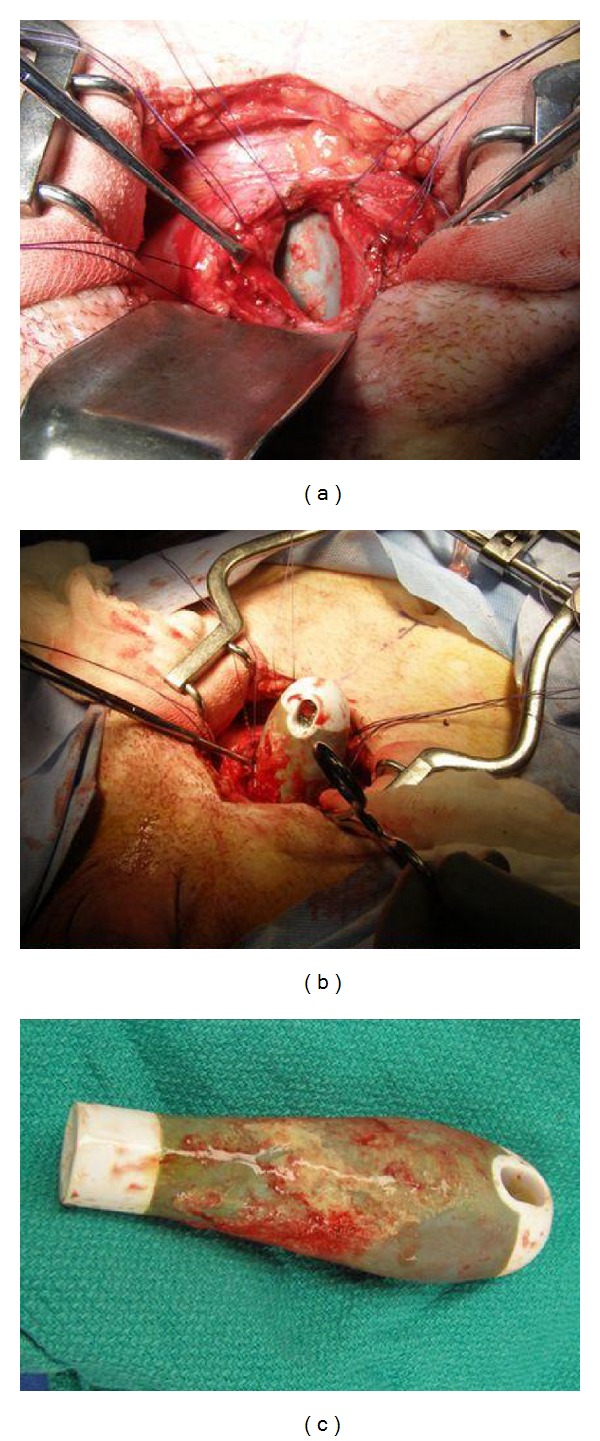
The toilet brush handle exposed through the cystotomy (a), partially extracted (b), and removed (c).

He was taken to the operating room where a further examination revealed an 8 cm deep defect in the perineum. He also had a bulbar urethral-cutaneous fistula (Figure 2). An open cystotomy was performed and the toilet brush handle was removed (Figure 3). 

He was treated with antibiotics and did well postoperatively. A psychiatric consult was completed and he was diagnosed with gender-identity disorder but was otherwise stable and safe to be discharged. The patient refused surgical repair of the fistula and the perineal defect.

## 3. Discussion

The previously mentioned patient poses a difficult clinical scenario. It is not surgically ideal to allow the persistence of the fistula. However, the patient's refusal of repair is consistent with some previous descriptions of UGUA [6, 7]. There is evidence to suggest that those who have been forced to participate in sexual acts against their will are more likely to participate in genital piercings, and by extension they may be more likely to perform other forms of urethral play [1]. There are no descriptions of UGUA in those who are abusers, however. Psychiatric disorders and intoxication have been related as causal in many cases of UGUA [2, 8]. Concordantly, our patient had unusual motivations and psychological abnormalities. However, Rinard et al. showed in their cross-sectional study that applying these attributes as a generalization of persons who perform UGUA is inaccurate [1]. 

The management of intravesical foreign bodies has been well described. Historically, perineal urethrostomy and/or open cystotomy have been performed. In the right clinical setting, that is, size of the object, endoscopic techniques are preferred and are often employed [2, 3, 9]. First, a thorough clinical evaluation is necessary for a patient presenting with a suspected genitourinary foreign body. Thereafter, pain and symptom control is the priority. Symptoms associated with intravesical foreign body include those of cystitis, dysuria frequency, and hematuria. More severe complications can arise and their consideration is paramount, for example, chronic or recurrent urinary tract infection, urinary retention, calcification, hydronephrosis, posterior urethral injury [10], obstructive uropathy, vesicovaginal fistula, squamous cell carcinoma, and sepsis [2, 3].

The urology subgroup of patients who perform UGUA are well known to nearly every practice. Understanding the contributing factors to this unusual behavior can aid the urologist in best managing their care. Psychosocial factors should be addressed after pain control, symptom management, and consideration of more significant complications. Endoscopic retrieval of the foreign object and genitourinary tract evaluation should be used in cases when feasible.
